# Complete Genome Sequence of Antarctic Bacterium *Psychrobacter* sp. Strain G

**DOI:** 10.1128/genomeA.00725-13

**Published:** 2013-09-19

**Authors:** Shuai Che, Lai Song, Weizhi Song, Meng Yang, Guiming Liu, Xuezheng Lin

**Affiliations:** Key Laboratory of Marine Bioactive Substances, the First Institute of Oceanography, SOA, Qingdao, Chinaa; CAS Key Laboratory of Genome Sciences and Information, Beijing Institute of Genomics, Chinese Academy of Sciences, Beijing, Chinab

## Abstract

Here, we report the complete genome sequence of *Psychrobacter* sp. strain G, isolated from King George Island, Antarctica, which can produce lipolytic enzymes at low temperatures. The genomics information of this strain will facilitate the study of the physiology, cold adaptation properties, and evolution of this genus.

## GENOME ANNOUNCEMENT

The genus *Psychrobacter* was described to accommodate Gram-negative, psychrotolerant, halotolerant, aerobic, nonmotile bacteria (1). *Psychrobacter* species have been isolated from a wide range of habitats, including Antarctic ornithogenic soils (2), sea ice (3), Siberian permafrost (4), marine crustaceans (5), clinical specimens (6), and seafood (7). *Psychrobacter* sp. strain G was collected from King George Island, Antarctica (62°12′39″S, 58°54′41″W), and it can produce extracellular lipolytic enzymes with activity at low temperatures (8).

The *Psychrobacter* sp. G genome was sequenced with 454 GS-FLX and Illumina platforms. A total of 157,071 high-quality 454 reads with an average of 371 bp were produced, providing about 17× coverage, while the Illumina reads provide about 454× coverage with mate-pair reads of 100 bp (insert size, ~3,000 bp). Primary assembly was performed with Newbler version 2.3 and then scaffolded with Illumina mate-pair reads using SSPACE (9). The gaps were closed with GapFiller (10) and PCR walking. Open reading frames were identified and annotated with the NCBI Prokaryotic Genome Automatic Annotation Pipeline (PGAAP) (11). The functions of genes were also annotated by Clusters of Orthologous Groups (COG) (12), KEGG (13), and InterProScan (14).

The complete genome of *Psychrobacter* sp. G consists of a circular chromosome of 3,079,438 bp (42.44% G+C content) plus 3 plasmids: PsyG_26 (26,087 bp), PsyG_4 (4,518 bp), and PsyG_3 (3,956 bp). The chromosome contains 2,614 protein-encoding genes (CDSs), 12 rRNA operons, and 48 tRNAs. A total of 1,967 of the CDSs were assigned to COGs, and 1,345 CDSs can be annotated into 165 pathways by using KAAS (15). The genome sequence enables the further study of the cold adaptation properties, lipolytic enzymes, and evolution of this genus.

### Nucleotide sequence accession numbers.

The genome sequence data have been deposited in GenBank under the accession no. CP006265 (chromosome), CP006266 (PsyG_26), CP006267 (PsyG_4), and CP006268 (PsyG_3).

## References

[1] Juni E Heym GA 1986 *Psychrobacter immobilis* gen. nov., sp. nov.: genospecies composed of gram-negative, aerobic, oxidase-positive coccobacilli. Int. J. Syst. Evol. Microbiol. 36:388–391

[2] Bowman JP Cavanagh J Austin JJ Sanderson K 1996 Novel *Psychrobacter* species from Antarctic ornithogenic soils. Int. J. Syst. Bacteriol. 46:841–848 8863407 10.1099/00207713-46-4-841

[3] Romanenko LA Lysenko AM Rohde M Mikhailov VV Stackebrandt E 2004 *Psychrobacter maritimus* sp. nov. and *Psychrobacter arenosus* sp. nov., isolated from coastal sea ice and sediments of the Sea of Japan. Int. J. Syst. Evol. Microbiol. 54:1741–1745 15388738 10.1099/ijs.0.63096-0

[4] Bakermans C Ayala-del-Río HL Ponder MA Vishnivetskaya T Gilichinsky D Thomashow MF Tiedje JM 2006 *Psychrobacter cryohalolentis* sp. nov. and *Psychrobacter arcticus* sp. nov., isolated from Siberian permafrost. Int. J. Syst. Evol. Microbiol. 56:1285–1291 16738105 10.1099/ijs.0.64043-0

[5] Romanenko LA Tanaka N Frolova GM Mikhailov VV 2009 *Psychrobacter fulvigenes* sp. nov., isolated from a marine crustacean from the Sea of Japan. Int. J. Syst. Evol. Microbiol. 59:1480–1486 19502339 10.1099/ijs.0.007195-0

[6] Wirth SE Ayala-del-Río HL Cole JA Kohlerschmidt DJ Musser KA Sepúlveda-Torres Ldel C Thompson LM Wolfgang WJ 2012 *Psychrobacter sanguinis* sp. nov., recovered from four clinical specimens over a 4-year period. Int. J. Syst. Evol. Microbiol. 62:49–54 21317274 10.1099/ijs.0.029058-0

[7] Jung SY Lee MH Oh TK Park YH Yoon JH 2005 *Psychrobacter cibarius* sp. nov., isolated from jeotgal, a traditional Korean fermented seafood. Int. J. Syst. Evol. Microbiol. 55:577–582 15774627 10.1099/ijs.0.63398-0

[8] Xuezheng L Shuoshuo C Guoying X Shuai W Ning D Jihong S 2010 Cloning and heterologous expression of two cold-active lipases from the Antarctic bacterium *Psychrobacter* sp. G. Polar Res. 29:421–429

[9] Boetzer M Henkel CV Jansen HJ Butler D Pirovano W 2011 Scaffolding pre-assembled contigs using SSPACE. Bioinformatics 27:578–579 21149342 10.1093/bioinformatics/btq683

[10] Boetzer M Pirovano W 2012 Toward almost closed genomes with GapFiller. Genome Biol. 13:R56. 10.1186/gb-2012-13-6-r56 22731987 PMC3446322

[11] Angiuoli SV Gussman A Klimke W Cochrane G Field D Garrity G Kodira CD Kyrpides N Madupu R Markowitz V Tatusova T Thom-son N White O 2008 Toward an online repository of Standard Operating Procedures (SOPs) for (meta)genomic annotation. Omics 12:137–141 18416670 10.1089/omi.2008.0017 PMC3196215

[12] Tatusov RL Fedorova ND Jackson JD Jacobs AR Kiryutin B Koonin EV Krylov DM Mazumder R Mekhedov SL Nikolskaya AN Rao BS Smirnov S Sverdlov AV Vasudevan S Wolf YI Yin JJ Natale DA 2003 The COG database: an updated version includes eukaryotes. BMC Bioinformatics 4:41. 10.1186/1471-2105-4-41 12969510 PMC222959

[13] Kanehisa M Araki M Goto S Hattori M Hirakawa M Itoh M Katayama T Kawashima S Okuda S Tokimatsu T Yamanishi Y 2008 KEGG for linking genomes to life and the environment. Nucleic Acids Res. 36:D480–D484. 10.1093/nar/gkm882 18077471 PMC2238879

[14] Zdobnov EM Apweiler R 2001 InterProScan—an integration platform for the signature-recognition methods in InterPro. Bioinformatics 17:847–848 11590104 10.1093/bioinformatics/17.9.847

[15] Moriya Y Itoh M Okuda S Yoshizawa AC Kanehisa M 2007 KAAS: an automatic genome annotation and pathway reconstruction server. Nucleic Acids Res. 35:W182–W185. 10.1093/nar/gkm321 17526522 PMC1933193

